# Transport of enniatin B and enniatin B1 across the blood-brain barrier and hints for neurotoxic effects in cerebral cells

**DOI:** 10.1371/journal.pone.0197406

**Published:** 2018-05-16

**Authors:** Isabel Krug, Matthias Behrens, Melanie Esselen, Hans-Ulrich Humpf

**Affiliations:** Institute of Food Chemistry, Westfälische Wilhelms-Universität Münster, Corrensstr. 45, Münster, Germany; Hungarian Academy of Sciences, HUNGARY

## Abstract

Enniatins are common contaminants of food and feed and belong to the group of the “emerging” mycotoxins, which are produced by various *Fusarium* species. Although a wide range of toxic effects, like antibacterial, antifungal, insecticidal and cytotoxic properties, have been described *in vitro*, so far, no cases of mycotoxicosis connected to enniatins *in vivo* are reported. Among this group of mycotoxins, enniatin B and enniatin B1 are the most prevalent compounds and therefore are present in the human diet. Enniatins can reach systemic circulation, thus, the investigation of possible neurotoxic effects is of importance. Different cerebral cells were used to address effects on cell death having an impact on the blood-brain barrier. The influence of enniatin B and enniatin B1 on cellular viability was examined via Cell Counting kit-8 assay (CCK-8) in three different cell types of the blood-brain barrier: porcine brain capillary endothelial cells (PBCEC), human brain microvascular endothelial cells (HBMEC) and human astrocytoma cells (CCF-STTG1). CCF-STTG1 cells were more sensitive to enniatin B (IC_50_ = 8.9 μM) and enniatin B1 (IC_50_ = 4.4 μM) than both endothelial cell types. In CCF-STTG1 cells, caspase-3 activation and lactate dehydrogenase (LDH) release were evaluated. Both compounds did not induce any LDH release and only enniatin B increased caspase-3 activity as a marker for apoptosis. The transport kinetics of enniatin B and enniatin B1 across the blood-brain barrier *in vitro* were evaluated using PBCEC, cultivated on Transwell^®^ filter inserts. Analysis of the apical and the basolateral compartment by high performance liquid chromatography-mass spectrometry revealed high influx rates for enniatin B and enniatin B1. Thus, both compounds can reach the brain parenchyma where neurotoxic effects cannot be ruled out.

## Introduction

Filamentous fungi can produce a broad range of (often) toxic secondary metabolites, called mycotoxins. Already at concentrations in the lower micromolar range these mycotoxins, can pose a potential health risk to humans and animals [1].

One group of mycotoxins which has drawn more and more attention over the last few years, is summarized as “emerging” mycotoxins. This group of toxins includes compounds which (not yet) are neither legally regulated nor routinely analyzed. In general, when talking about “emerging” mycotoxins, this relates mainly to secondary fungal metabolites produced by various *Fusarium* species, like beauvericin (BEA), moniliformin (MON), fusaproliferin (FUS) and enniatins (ENNs) [1, 2].

**Fig 1:**
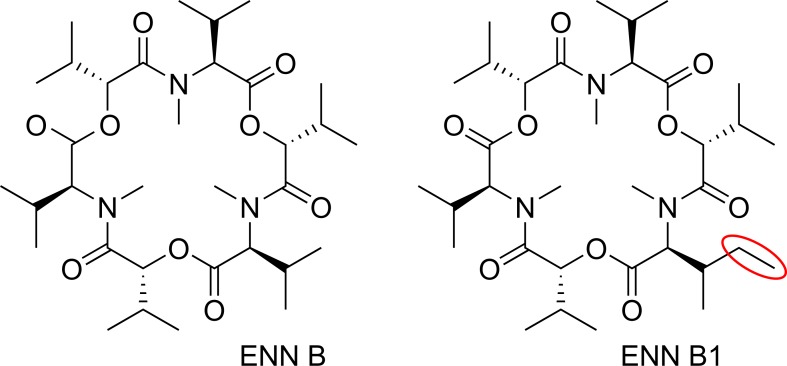
Structures of ENN B and ENN B1.

The most prevalent toxins of the “emerging” mycotoxins are the ENNs. Up to now, 29 structural analogues, which are mainly produced by *Fusarium* species, are described in literature. Regarding their structural properties, ENNs are cyclic hexadepsipeptides, which are produced non-ribosomally via the enniatin synthetase [3]. They consist of alternating d-2-hydroxyisovaleric acids and *N*-methyl-l-amino acids. Peptide bonds and intramolecular ester (lactone) bonds link the subunits forming an 18-membered ring [4]. In particular, enniatin B (ENN B) and enniatin B1 (ENN B1) are of central interest as they are the most prevalent compounds of this group of mycotoxins and occur in relatively high concentrations in *Fusarium* contaminated food and feed. For example, in grain concentrations up to 5.8 mg/kg ENN B in Norwegian wheat and 18.3 mg/kg ENN B1 in Finnish spring wheat were found [5]. Thus, this study focusses on the compounds ENN B and ENN B1 (Fig 1), as representatives of the group of enniatins. They only differ in one methyl group as highlighted in Fig 1.

Remarkably, due to their pharmacologic properties a mixture of the enniatins ENN B, B1, A and A1, found application as local antibiotic (marketed under the name “fusafungine”) to treat upper respiratory tract infections [6]. Though, because of reported severe allergic reactions to these remedies, the admission to the European market is currently under revision [7].

Systemic exposure of humans to ENN B and ENN B1 is described as these mycotoxins are detected in blood, urine and breast milk [8–10]. In various *in vivo*, *in vitro* as well as *ex vivo-*studies, it was shown, that cyclic hexadepsipeptides, like ENNs, are able to cross barriers of mammalian organisms very fast and to a high extent. Therefore, they can easily reach systemic circulation. ENNs are able to cross an *ex vivo* human skin barrier model to a great amount with ENN B showing the highest permeation (k_p,v_ = 9.44 × 10^−6^ cm/h) [11]. Taevenier et al. [12] showed that ENNs could penetrate porcine buccal mucosa with steady-state plasma concentrations up to 1.3 mg/L [12]. In a Caco-2 barrier experiment the absorption after 4 h exposure was > 65% for ENN B and ENN B1, also the duodenal bioavailability *in vitro* (Caco-2 model) was > 50% after 48 h for both toxins [13].

So far, one single study on the permeation of ENNs and BEA across the blood-brain barrier (BBB) in mice was performed. This study was conducted over the course of 100 minutes. The results of this short time *in vivo-*mouse study, indicate a high and rapid influx into the brain with distribution to the brain parenchyma [14].Our study aims to add more insights to the kinetics of the transfer of ENNs with a longer exposure time (48 h) and eight sampling times over the course of the experiment. Thus, a better kinetic profile can be shown.

It is known, that several other fungal toxins, like ochratoxin A, T-2 and HT-2 toxins or deoxinivalenol, are able to cross the BBB and affect the viability and functions of brain cells, including astrocytes, microglia and endothelial cells [15–18]. Considered that ENNs reach systemic circulation, the permeation properties through the BBB are of crucial importance. The passage of ENNs through the BBB could cause neurotoxic effects in cells of the central nervous system.

The BBB is a safety feature protecting highly sensitive neuronal cells of the brain from adverse effects. In general, the BBB consists of three different cell types, which together form a neurovascular unit: Endothelial cells, astrocytes and pericytes [19]. The BBB is a highly selective interface which limits the unhindered interaction of substances (endogenous or exogenous) from systemic circulation with the brain. To estimate the probability of a compound to reach the brain, different approaches are applicable [20, 21]. In this study, a well-established cell culture model based on primary porcine brain capillary endothelial cells (PBCEC) was used. This model combines many advantages compared to other systems. In contrast to *in vivo-* and *ex vivo-*experiments, there is no need for invasive procedures and interindividual differences can be reduced. Compared to other *in vitro*-models relying on continuous cell lines, the handling and costs might be higher, but morphological features of the brain endothelium are much better reflected in primary cells [20]. Even in monoculture, with PBCECs, high TEER (transendothelial electrical resistance) values of 1000 Ω·cm^2^ and higher can be reached. Such TEER values are often not attained by other *in vitro-*cell culture models that are applied for transport studies. This property of the PBCEC is close to the *in vivo*-situation and ensures the tightness and reliability of a barrier system to study transport characteristics [16, 20, 22]. In order to assess the possibility of ENNs reaching the brain, the transport kinetics of ENN B and B1 were evaluated using this *in vitro*-cell culture system based on PBCEC mimicking the BBB.

As mentioned above, the BBB consists of different cell types. Thus, as soon as ENNs are able to cross the BBB it becomes relevant to investigate possible (adverse) effects of ENNs on different cells of the BBB. For that matter an astrocytoma cell line (CCF-STTG1 cells) and, besides the primary cells (PBCEC), which are not suited for all of the applied test systems, human brain microvascular endothelial cells (HBMEC) were included in the evaluations. Different endpoints concerning cell death like cytotoxicity, caspase-3 activity and LDH leakage were considered.

## Material and methods

### Chemicals and reagents

All chemicals for ENN quantitation were purchased at VWR International GmbH (Darmstadt, Germany), Grüssing GmbH Analytica (Filsum, Germany) and Sigma-Aldrich GmbH (Steinheim, Germany). Cell culture media and supplements were purchased at Biochrom AG (Berlin, Germany) and PAN-Biotech GmbH (Aidenbach, Germany). Hydrocortisone, bicinchoninic acid (BCA) and bovine serum albumin (BSA) were obtained from Sigma-Aldrich (Steinheim, Germany). Purified water was retrieved from a Purelab Flex 2 purification system (Veolia Water Technologies, Celle, Germany). ENN B (E4511) and ENN B1 (E5286) with purities ≥ 95% were purchased from Sigma-Aldrich GmbH (Steinheim, Germany). For experiments, separate stock solutions of 1 mM ENN B and B1 in acetonitrile (ACN) were prepared and stored at –20°C.

### Effects of ENN B and ENN B1 on cell death

#### Viability assay in cell lines (HBMEC and CCF-STTG1)

To test the viability and to define the working range for later transport studies ENN B/ENN B1 stock solutions in ACN were diluted to the respective concentrations with serum free culture medium (1% ACN). An astrocytoma cell line, CCF-STTG1 (ATCC, Manassas, USA), and HBMEC cell line (human brain microvascular endothelial cells, kindly provided by Prof. Dr. Karch, Institute of Hygiene, University of Muenster (WWU Muenster)) were cultivated in RPMI 1640 medium supplemented with 100 U/mL penicillin, 100 μg/mL streptomycin, 2 mM l-glutamine and 10% (v/v) fetal calf serum (FCS) (medium for HBMEC additionally supplemented with 1 mM Na-pyruvate) using standardized culture conditions (37°C, 5% CO_2_, saturated humidified atmosphere). Culture medium was changed every 2–3 d, and the cells were subcultivated after trypsination when they reached a microscopic confluence of approximately 80%. For the evaluation of cytotoxic effects of ENN B and B1 in CCF-STTG1 and HBMEC, Cell Counting Kit-8 (CCK-8, Dojindo Laboratories, Tokyo Japan) was applied. The assay was performed according to the manufacturer’s instructions with slight modifications.

Briefly, the cells were seeded in 96-well tissue culture plates in a density of 1 × 10^4^ cells/well (CCF-STTG1) and 4 × 10^3^ cells/well (HBMEC), respectively. The cells were allowed to grow for 24 h, then the culture medium was replaced by serum free medium (100 μL/well), and the cells were cultivated for another 24 h (37°C, 5% CO_2_, saturated humidified atmosphere) before treatment. The cells were treated with ENN B and ENN B1 in a concentration range from 0.1 μM to 10 μM and were incubated for 48 h. After toxin exposure, the toxin solution was exchanged with a dye solution WST-8 (CCK-8 solution, water soluble tetrazolium salt-8, 2-(2-methoxy-4-nitrophenyl)-3-)4-nitrophenyl)-5-(2,4-disulfophenyl)-2*H*-tetrazolium monosodium salt), 1:10 diluted with fresh serum free medium, which was added to the cells followed by incubation for 60 min at 37°C. WST-8 dye is reduced to a water-soluble formazan by cellular dehydrogenases of viable cells, which increases the absorbance at λ = 457 nm and was measured with an Infinite M200 PRO microplate reader with Tecan i-control software version 1.7.1.12 (Tecan, Crailsheim, Germany). The amount of formazan generated by the activity of dehydrogenases in cells is directly proportional to the number of viable cells per well. The results are normalized to a solvent treated negative control (1% ACN).

#### Viability assay in primary porcine brain capillary endothelial cells (PBCEC)

Primary porcine brain capillary endothelial cells (PBCEC) were kindly provided by the group of Prof. Dr. Langer, Institute of Pharmaceutical Technology, WWU Münster. Cellular viability was tested after treatment for 48 h using the CCK-8 assay, like applied for the cell lines but with minor modifications. Based on earlier protocols [16, 22] for the cultivation of PBCEC, the cryopreserved PBCEC were thawed at 37°C in a water bath, gently resuspended in culture medium (Medium 199 Earle’s with 100 U/mL penicillin, 100 μg/mL streptomycin, 100 μg/mL gentamycin, 4.1 mM l-glutamine and 10% fetal calf serum) and centrifugated at 220 × *g* at 20°C for 10 min. After removal of the supernatant, fresh complete medium was added and the cell pellet was resuspended twice and diluted to desired cell density. The cells were seeded on rat tail collagen coated 96-well tissue culture plates with 100 μL of the cell suspension per well. PBCEC were allowed to grow for 48 h at 37°C, 5% CO_2_ under saturated humidified conditions. Complete medium was exchanged for serum free medium (DMEM/Ham’s F-12 1:1 with 100 U/mL penicillin, 100 μg/mL streptomycin, 100 μg/mL gentamycin, 0.7 mM l-glutamine and 550 nM hydrocortisone (Sigma-Aldrich, Steinheim, Germany)). On day 5 in culture (96 h after seeding) half of the medium (50 μL) was removed and exchanged with 50 μL double concentrated ENN solution to reach the desired final concentrations in a concentration range from 0.1 μM to 10 μM. The cells were treated with the test substances in concentrations from 0.1 to 10 μM for 48 h. Due to the sensitivity of the primary cells, after completion of 48 h, the incubation medium was not exchanged but each well was supplemented with 10 μL of the CCK-8 solution and incubated for 70 min at 37°C, 5% CO_2_ in a saturated humidified atmosphere. Then the absorbance of the generated formazan dye was measured at 457 nm with 650 nm as reference wavelength. After subtracting the reference absorption as well as a blank absorption without cells, the viabilities were normalized to a solvent treated negative control (1% ACN).

#### Apoptosis: Caspase-3 activity

For studying caspase-3 activity CCF-STTG1 cells were seeded in 12 well tissue culture plates in a cell density of 2.25 × 10^5^ cells/well. The cells were grown for 24 h until a confluence of at least 70% was reached, then the medium was changed to serum free medium (RPMI 1640 medium supplemented with 100 U/mL penicillin, 100 μg/mL streptomycin and 2 mM l-glutamine). Concentrations from 0.1 μM to 2.5 μM were chosen to prevent strong cytotoxic effects, which might interfere with the endpoint of interest. 24 h after adaption to serum free conditions, cells were incubated with ENN B and ENN B1. Caspase-3 activity was determined in lysates. To prepare the lysates, the incubation medium was removed (used in LDH leakage assay) and the cell layer was rinsed with 200 μL phosphate-buffered saline without magnesium and calcium (PBS^-/-^) twice. Followed by addition of 120 μL lysis buffer (ice cold) (10 mM TRIS, 100 mM NaCl, 1 mM EDTA and 1% Triton-X-100 in water) and lysis for 15 min on ice. Lysed cells were scraped off, transferred to microreaction tubes and centrifuged (10 000 × *g*, 4°C, 10 min). 30 μL of the supernatant were subsequently used for the measurement. 33 μL reaction solution were added to 30 μL of the supernatant and incubated for 1 and 2 h at 37°C, 5% CO_2_ and saturated humidified atmosphere and then measured at λ_ex_ = 405 nm and λ_em_ = 520 nm (Infinite M200 PRO microplate reader). Reaction solution comprised 2 parts reaction buffer (50 mM PIPES, 12.7 mM EDTA, 8.1 mM CHAPS ad 100 mL with water, pH 7.4), 3 parts water, 0.5 parts caspase-substrate (1 mM Ac-DEVD-AFC, Sigma-Aldrich GmbH, Seelze, Germany) and 0.05 parts DTT solution (1 M). The results were calculated as μmol AFC/μg protein and normalized to the solvent treated control (ACN 0.25%).

For quantitation of the total protein content a bicinchoninic acid (BCA) assay kit (Sigma-Aldrich GmbH, Seelze, Germany) was applied. Bovine serum albumin (BSA) served as standard for external calibration. 15 μL of cell lysates were diluted with 200 μL BCA reagent (BCA solution/4% copper sulfate, 50+1, v/v) in a 96-well tissue culture plate and incubated for 30 min at 37°C. After that the absorbance was measured at 560 nm with a Infinite M200 PRO microplate reader (Tecan, Crailsheim, Germany).

#### Necrotic cell death: Lactate dehydrogenase (LDH) leakage

For measurement of lactate dehydrogenase (LDH) leakage, cell lysates and treatment solutions (serum free medium with respective ENN B/B1 content) after incubation for 48 h were used. The cell lysates were prepared as described for caspase-3 activation assay. To determine the LDH activity, 15 μL of the lysate and 40 μL of the incubation medium were each added to 200 μL substrate buffer (100 mM HEPES, 10 mM sodium pyruvate and 0.21 mM NADH, adjusted to pH 7.0), incubated over the course of 30 min at 37°C and absorption was measured every 2 min at 355 nm in a Infinite M200 PRO microplate reader (Tecan, Crailsheim, Germany).

### Transfer studies

In accordance with the thawing and seeding procedure for PBCEC, described for the viability test, 500 μL PBCEC suspension (2.5 × 10^5^ cells/well) in culture medium were seeded on rat tail collagen coated 12-well Transwell^®^ microporous polycarbonate filter inserts (Corning, Wiesbaden, Germany; growth area 1.12 cm^2^, pore size 0.4 μm) in the upper (apical) compartment of the filter system. The lower (basolateral) compartment was filled with 1500 μL complete medium. This two-compartment system was used as model system for the BBB with the apical compartment reflecting the “blood”-side and the basolateral compartment serving as the “brain”-side. After 48 h, the complete medium was replaced by serum free medium. Additionally, 550 nM hydrocortisone (Sigma-Aldrich, Steinheim, Germany) was supplemented to urge differentiation of cells. After another 48 h, experiments studying barrier integrity and permeability were performed.

Before starting each experiment, the TEER values and the electrical capacitance of all cell layers were measured via cellular impedance spectroscopy (cellZscope^®^, nanoAnalytics, Münster, Germany). Only Transwell^®^ filters with cell layers possessing TEER values of more than 300 Ω·cm^2^ and electrical capacitances (C_Cl_) of 0.4 to 0.6 μF/cm^2^ were used for transfer studies. TEER values relate to the constitution of the tight junctions limiting paracellular transfer of compounds. In addition to the TEER, electrical capacitances (C_Cl_) were monitored for 48 h. Cellular phospholipid bilayers act like an electrical capacitor and also contribute to the cellular impedance. Changes of the C_Cl_ value give information about the integrity of the cell monolayer [23]. In this experiment, a parallel set up was applied. For each treatment, one triplicate of filters was monitored via cellular impedance spectroscopy over the course of 48 h using a cellZscope^®^ device–here, the basolateral compartment was prepared with 1650 μL serum free medium and the volume in the apical compartment was filled to 760 μL (ratio 1:2.17). Another triplicate for the same incubation conditions was kept in Transwell^®^ filter cell culture multiple well plates (12 well format) over the course of 48 h. In the multiple well plates the ratio between the apical and the basolateral compartment was 1:3, therefore the starting volume in the apical compartment is 500 μL and 1500 μL in the basolateral compartment. Here, samples for quantitation of transfer of enniatins across the barrier were drawn after 1, 2.5, 6.5, 18, 24, 28, 42 and 48 hours in this given ratio i.e. 30 μL apical and 90 μL basolateral. After 48 h, the final TEER values of the filters from the multiple well plate were measured to ensure the stability of the cell layer throughout the experiment. To double check the integrity of the barrier, lucifer yellow (LY, 50 μM), as a paracellular transfer marker, was applied. The amount of LY found in the basolateral compartment after 1 h was measured at λ_ex_ = 430 nm and λ _em_ = 540 nm.

After the completion of the experiment, the polycarbonate filter membranes were extracted. The filters were cut out and extracted with ACN/H_2_O (8+2, v/v) via ultrasonication for 1 h. The extract was evaporated (vacuum concentrator BA-VC-200 H, Bachofer GmbH + Co KG, Reutlingen, Germany) and reconstituted in 200 μL of ACN/H_2_O (8+2, v/v). The obtained samples and filter extracts were analyzed and quantitated with high performance liquid chromatography-tandem mass spectrometry (HPLC-MS/MS).

### Quantitation of ENN B and ENN B1

The quantitation of ENN B and ENN B1 by HPLC-MS/MS was carried out using an 1100 series (Agilent Technologies, Santa Clara, USA) high performance liquid chromatography (HPLC) system and API 3200 (AB SCIEX Germany GmbH, Darmstadt, Germany) mass spectrometer. Both devices were operated with Analyst Version 1.6.2 software (AB SCIEX Germany GmbH, Darmstadt, Germany). A Synergi 4 μm Fusion-RP 80 Å 50 × 2.0 mm (Phenomenex) equipped with a Synergy 4 μm Fusion-RP 4 × 2.0 mm (Phenomenex) precolumn was used and maintained at 40°C. A binary gradient consisting of ACN (A) and water (B) (both containing 0.1% formic acid) was applied. The concentration of the analytes was determined directly in cell culture medium and quantitated using a matrix-matched external calibration in serum free cell culture medium (0.1% hydrocortisone), incubated with PBCEC for 48 h. 10 μL of sample solution were injected. With a flow rate of 500 μL/min the starting condition was 20% A, which was held for 2 min. The percentage of A was increased to 100% within 3 min and kept constant for further 1.5 min. Starting at 6.5 min the gradient was decreased from 100% A to the starting conditions (20% A) and held for 1.5 min. The parameters of the mass spectrometer (API 3200) were set to: Curtain gas 30 psi N_2_, source temperature 400°C, nebulizer gas (GS1) 35 psi N_2_ and heater gas (GS2) and 45 psi respectively, and a dwell time of 25 ms per MRM transition was used. Electrospray ionization in positive ionization mode was applied with the ion spray voltage set to 5500 V. The two multiple reaction monitoring (MRM) transitions, which possessed the best signal-to-noise ratios were used for analysis of the two compounds (ENN B and ENN B1), the one with the highest signal intensity was chosen as quantifier. Table 1 lists the corresponding HPLC-MS/MS parameters for both ENNs.

**Table 1:** Parameters for multiple reaction monitoring (MRM) for the two analytes.

Analyte	Q1 mass [*m/z*]	Q3 mass [*m/z*]	DP [V]	CE [V]	CXP [V]
ENN B	640.5 [M + H]^+^	214^a^ 196^b^	71 71	55 31	5 5
ENN B1	654.4 [M + H]^+^	210^a^ 196^b^	58 58	30 33	17 16

^a^ quantifier

^b^ qualifier

### Permeability calculations

Time independent permeability coefficients were derived, to enable the comparison of permeation of different compounds through the BBB. The permeability coefficients *P* were calculated according to Eq 1 [16]:

$$P\;\left[\frac{\mathrm{cm}}{s}\right]=\frac{c_{bas}[\%]}{c_{0h,\;ap}[\%]}∙\frac{V_{ap}[cm^{3}]}{A\;[cm^{2}]∙t\;[s]}$$ (1)

*c*_*bas*_ represents the amount of test substance, which is found in the basolateral compartment at a distinct timepoint *t*. *V*_*ap*_ with *c*_*0h*, *ap*_ describe the initial volume of the apical (“blood”) compartment and applied concentration in the apical compartment at the start of the experiment, respectively. The filter surface of the Transwell^®^ filter insert is presented as *A*. To exclude hindrance of passage by the polycarbonate filter membrane, a cell free experiment was carried out. ENN B and ENN B1 were applied at 10 μM concentrations in the apical compartment of a rat tail collagen coated filter insert. *P*_*e*,_ the permeability of the mycotoxin across the cell monolayer, corrected for the filter permeability, was calculated according to Eq 2.

$$\frac{1}{P_{e}[\frac{\mathrm{cm}}{s}]}=\frac{1}{P_{c+f}[\frac{\mathrm{cm}}{s}]}‑\frac{1}{P_{f}[\frac{\mathrm{cm}}{s}]}$$ (2)

The permeability of the mycotoxins in the cell free experiment *P*_*f*_ is included in the calculation of the permeability coefficient of the study (*P*_*c+f*_) as shown in Eq 2.

### Statistical analysis

Cellular viability studies were performed as a minimum in triplicates with cells from at least three independent passages/preparations (*n* ≥ 9, technical replicates). Caspase-3 assay and LDH leakage assay were performed in duplicates from at least three independent experiments (*n* ≥ 6, technical replicates). The data are presented as the mean ± standard deviation (SD). The significance indicated refers to the significance level as compared to the solvent treated control calculated with one-way ANOVA and Dunnett’s multiple-comparison test via OriginPro 2016G (64-bit) Sr2 b9.3.2.303 (Significance levels: * low significant (p ≤ 0.05); ** medium significant (p ≤ 0.01); ***, highly significant (p ≤ 0.001)). For transfer experiments PBCEC from at least three different preparations were used and the transport experiments were performed in triplicates (*n* = 9). The data are presented as the mean ± standard deviation (SD).

## Results and discussion

In this study, effects of ENN B and ENN B1 on cell death in different cells types, involved in the formation of the BBB, were evaluated. In a further step, transport properties of ENNs across a primary porcine BBB model were investigated.

### Effects of ENN B and ENN B1 on cell death

#### Cell viability

**Fig 2:**
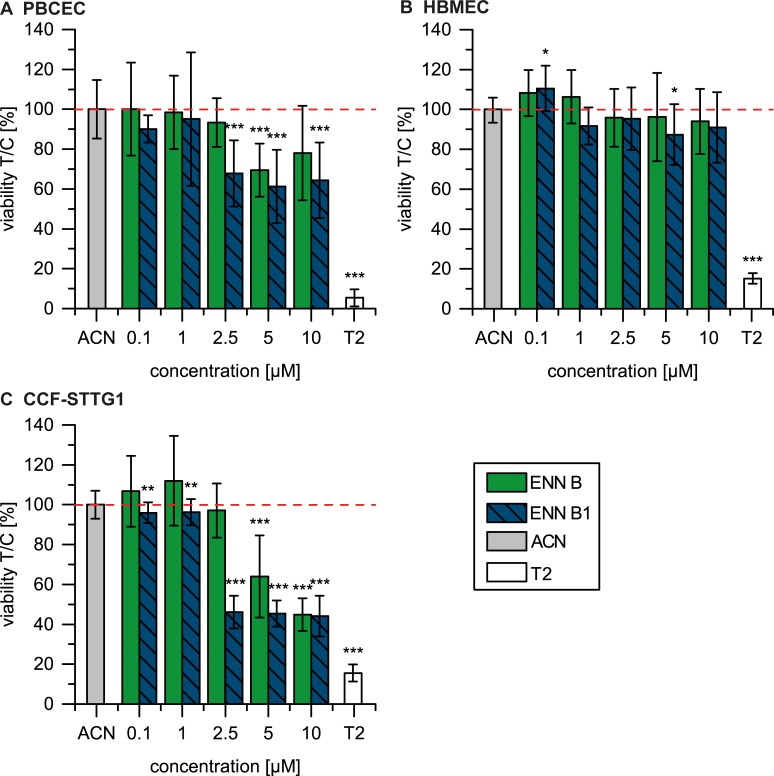
**Viability of PBCEC (*n* ≥ 9) (A), HBMEC (*n* ≥ 9) (B) and CCF-STTG1 cells (*n* ≥ 9) (C) after treatment with ENN B and ENN B1 for 48 h.** As positive control T-2 toxin (T2, 10 μM) was applied. Data are presented as the mean ± standard deviation (SD). The significance indicated refers to the significance level as compared to the solvent treated control (1% ACN) calculated with one-way ANOVA and Dunnett’s multiple-comparison (Significance levels: * low significant (p ≤ 0.05); ** moderately significant (p ≤ 0.01); *** highly significant (p ≤ 0.001)).

To determine and compare possible effects of ENN B and ENN B1 on cellular viability in different cells of the BBB, a cytotoxicity assay (CCK-8 assay), was carried out using primary porcine brain capillary endothelial cells (PBCEC), human brain microvascular capillary cells (HBMEC, passages 21, 22, 35 and 44) and CCF-STTG1 (passages 58 – 61), an astrocytoma cell line. The CCK-8 assay is based on the reduction of a water-soluble tetrazolium salt-8 (WST-8) dye by cellular dehydrogenases of viable cells to the corresponding formazan which is directly proportional to the number of metabolically active cells. Cells were incubated with the test compounds in concentrations ranging from 0.1 μM to 10 μM for 48 h. The concentrations in the experimental setup were chosen to cover the broad variations of contamination levels with ENNs found in food and feed. Annual contamination levels vary from ~ 10 μg/kg up to concentrations of 5.8 mg/kg ENN B and 18.3 mg/kg ENN B1 [5]. In a pig-feeding study, after oral intake of 0.05 mg/kg b.w. ENNs, reflecting a 1 mg/kg contamination of feed, concentrations in pig plasma were 0.073 μg/mL (ENN B, 0.114 μM) and 0.035 μg/mL (ENN B1, 0.054 μM) [24]. In a worst case scenario with 5.8 mg/kg ENN B [5] this would correlate to theoretical plasma levels of 0.423 μg/mL (0.662 μM) and 18.3 mg/kg ENN B1 [5] would result in 0.641 μg/mL (0.980 μM), respectively. In a worst case acute intoxication our chosen concentration range may be relevant. In Fig 2 the results of the viability assay are summarized.

The treatment of the porcine and human brain capillary endothelial cells lead to different results. While in the continuous cell line, HBMEC, no effect on viability compared to the solvent control, was observed at the concentration range from 0.1 μM to 10 μM (Fig 2B), viability of the primary cells, PBCEC (Fig 2A), was decreased after treatment with ENN B and ENN B1. ENN B was statistically highly significantly cytotoxic at concentrations above 5 μM with a reduction of cellular viability to a minimum of 70%. The exposure of PBCEC to ENN B1 resulted in a slightly higher cytotoxic effect. Cell viability was highly significantly inhibited at concentrations of 2.5 μM and higher, with reduction to 64% relative viability at 10 μM.

The effects on viability caused by ENN B and ENN B1 in endothelial cells of the brain, were less pronounced than the effects observed in the astrocytoma cell line, CCF-STTG1 (Fig 2C). In CCF-STTG1 cells, ENN B and ENN B1 caused considerably stronger effects on cellular viability. Here, ENN B presented highly significant cytotoxic effects at 5 μM and 10 μM. After incubation with ENN B1 weak but significant effects were observed already at 0.1 μM and 1 μM, strong and highly significant cytotoxic effects were detected at 2.5 μM and higher. From these results IC_50_ values for ENN B of 8.9 μM and ENN B1 of 4.4 μM in CCF-STTG1 cells were calculated.

In general, a higher cytotoxic potential was observed for ENN B1 in all three cell types. These results are in good accordance to most results found in literature, concerning the cytotoxicity of ENNs.

Wätjen et al. [25] describe cytotoxic effects of ENNs in different tumor cell lines (H4IIE rat hepatoma, HepG2 human hepatoma and C6 rat glioma cells) applying the MTT-assay. In C6 glioma and HepG2 hepatoma cells IC_50_ values range between 2.5 and 10 μM, respectively. The strongest cytotoxic effect is evident in H4IIE cells with IC_50_ values between 1 and 1.5 μM [19]. Meca et al. [26] discuss that almost all ENNs they tested (A, A1, A2, B, B1, B2, B4 and J3) exert cytotoxic effects in HepG2, HT-29 (human colorectal adenocarcinoma cell line) and Caco-2 cells (human colorectal adenocarcinoma cell line) in a dose-dependent manner. After a 48 h incubation ENN B leads to an IC_50_ value of 2.8 μM and ENN B1 to 3.7 μM in HT-29 cells. For the other cell lines, HepG2 and Caco-2, treatment with ENN B1 lead to IC_50_ values of 8.5 μM and 11.5 μM, respectively, whereas no IC_50_ could be calculated for ENN B [26]. Ivanova et al. [27] describe IC_50_ values of ENN B and B1 after 24 h incubation (Alamar Blue^™^ assay). For MRC-5 cells (human lung fibroblast cell line) and HepG2 cells, ENN B shows IC_50_ values of 9.8 μM and 435.9 μM, respectively. For ENN B1 IC_50_ values of 4.7 μM (MRC-5) and 36 μM (HepG2) are reported [27]. In general, ENN B1 exhibits higher cytotoxic properties. The comparison of the cytotoxic potential (MTT-assay) of a mixture of ENNs in 27 different cell models, after a longer exposure time (72 h), concludes that the IC_50_ for most tumor cell models is lower (<5 μM) than for normal cell lines (e.g. endothelial cell model (HUVEC)) [28]. This also supports the observation in our experiments, where CCF-STTG1 cells, an astrocytoma cell line, was considerably more sensitive to ENN B and B1 than normal cell types, like PBCEC or HBMEC. Tumor cells in general are faster dividing cells with higher proliferation than other cell types. Taking cell cycle arrests in the G0/G1 phase caused by ENNs into account [28], the assumption that ENNs might have an impact on apoptotic signaling pathways associated with cell proliferation can be made. This considered, it is likely that the impact of ENNs on proliferation and cellular viability is higher in tumor cell types. Therefore, the cytotoxicity observed in our study can be ranked as follows ENN B1 > ENN B and the sensitivity of cell types: CCF-STTG1 > PBCEC > HBMEC.

As the astrocytoma cell line (CCF-STTG1) was substantially more sensitive to the exposure to ENN B and ENN B1, further experiments concerning the type of cell death were carried out with these cells.

#### Apoptosis and necrosis: Caspase-3 activation and LDH leakage

**Fig 3:**
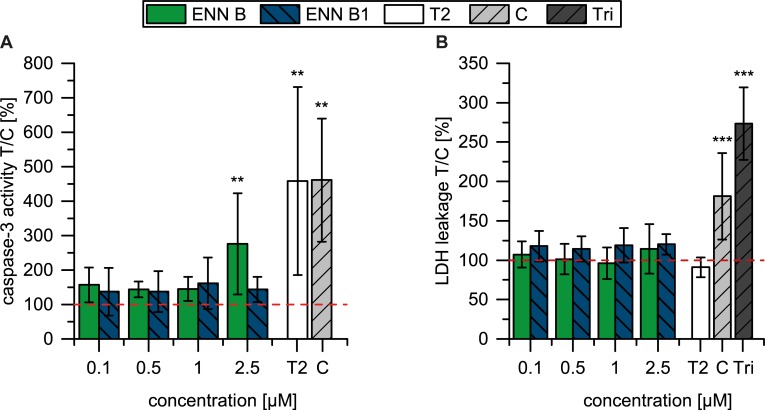
**Caspase-3 activation in CCF-STTG1 after incubation with ENN B and ENN B1 for 48 h (*n* ≥ 6) (A). LDH leakage in CCF-STTG1 after incubation with ENN B and ENN B1 for 48 h (*n* ≥ 6) (B).** Positive controls are T-2 toxin (T2, 10 μM), camptothecin (C, 10 μM) and Triton X-100 (Tri, 0.1%). Data are presented as the mean ± standard deviation (SD). The significance indicated refers to the significance level as compared to the solvent treated control (0.25% ACN) calculated with one-way ANOVA and Dunnett’s multiple-comparison (Significance levels: * low significant (p ≤ 0.05); ** moderately significant (p ≤ 0.01); *** highly significant (p ≤ 0.001)).

To distinguish whether the cell death in CCF-STTG1 cells was of apoptotic or necrotic nature, the caspase-3 activation, as indicator for the programmed cell death, and LDH leakage, as marker for necrotic cell death, were considered. For evaluations concerning the programmed cell death, CCF-STTG1 cells were incubated with ENN B and ENN B1 for 48 h with concentrations from 0.1 μM to 2.5 μM, where first cytotoxic effects were observed. For ENN B a 2.7-fold, highly significant increase of caspase-3 activity compared to the solvent control was shown for the treatment with 2.5 μM ENN B for 48 h. Only minor differences in comparison to the solvent control were measured for ENN B1 with a maximum increase of caspase-3 activity to 160% at 1 μM (Fig 3A).

Concerning LDH release as a marker for necrotic cell death, slightly higher LDH leakage to the medium was observed for the cells treated with ENN B1 (Fig 3B), but overall no significant increase of LDH release was detectable for ENN B and ENN B1.

As reported by Wätjen et al. [25] ENN B and ENN B1 induces apoptosis in hepatoma cells in low micromolar concentrations of 0.5 and 1 μM after 24 h, measured via the caspase-3/7 activation and nuclear fragmentation. In contrast to this, after 48 h incubation, our results suggest a stronger caspase-3 activation caused by ENN B. A significant increase of caspase-3/7 activity after treatment with both, ENN B and ENN B1, with a higher caspase-3 activity for cells treated with ENN B1 is described [25]. Dornetshuber et al. [22] also confirm no signs of LDH leakage caused by a mixture of ENNs, whereas already the minimally active concentration of 2.5 μM ENNs prompts cell cycle arrests and at higher concentration (10 μM ENNs) a high activation of caspase-3/7 is resulting [28].

To sum this up, no strong differences of the effects of ENN B and ENN B1 were expected because their chemical structures and properties are very similar. ENN B induces lower cytotoxicity but an induction of caspase-3 activity. This could indicate different modes of action of ENN B and ENN B1, where ENN B is rather initiating signalling pathways and does not necessarily exert high acute cytotoxicity, whereas ENN B1 is more cytotoxic and will earlier lead to necrotic cell death. But further investigations in terms of different time points, concentrations and considered endpoints are required to investigate differences in ENNs toxicodynamics.

### Transfer of ENN B and ENN B1 across a porcine BBB model

To evaluate whether ENN B and ENN B1 could cross the BBB and subsequently cause neurotoxic effects in the brain parenchyma, an *in vitro-*model using PBCEC mimicking the BBB was applied.

**Fig 4:**
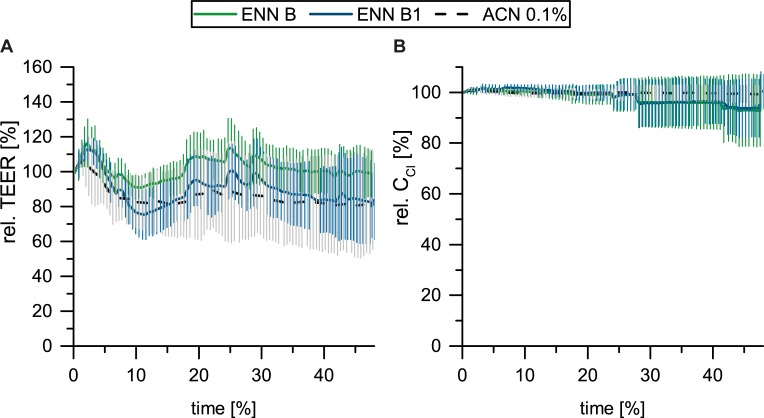
**TEER values of PBCEC, treated with 1 μM ENN B and ENN B1 for 48 h (*n* > 6, negative control ACN 0.1%) (A) and C**_**Cl**_**-measurement (B) of PBCEC incubated with 1 μM ENN B and ENN B1 (*n* > 6, negative control ACN 0.1%) over the course of 48 h.** Data are presented as the mean ± standard deviation (SD). The significance indicated refers to the significance level as compared to the solvent treated control (0.1% ACN) calculated with one-way ANOVA and Dunnett’s multiple-comparison (Significance levels: * significant (p ≤ 0.05); ** highly significant (p ≤ 0.01); *** very highly significant (p ≤ 0.001)).

For passive transport (apical → basolateral) studies PBCEC cells were incubated with 1 μM concentrations (= 0.5 nmol) of either ENN B or ENN B1 in the apical (upper) compartment. 1 μM was chosen, because this concentration did not show any cytotoxic effects in PBCEC over the course of 48 h (Fig 2A). Furthermore, it was shown that this concentration does not lead to a breakdown of the TEER value (Fig 4A) and does not impair PBCEC confluency of the monolayer of the barrier-forming endothelial cells reflected by capacitance *C*_*Cl*_ (Fig 4B).

**Fig 5:**
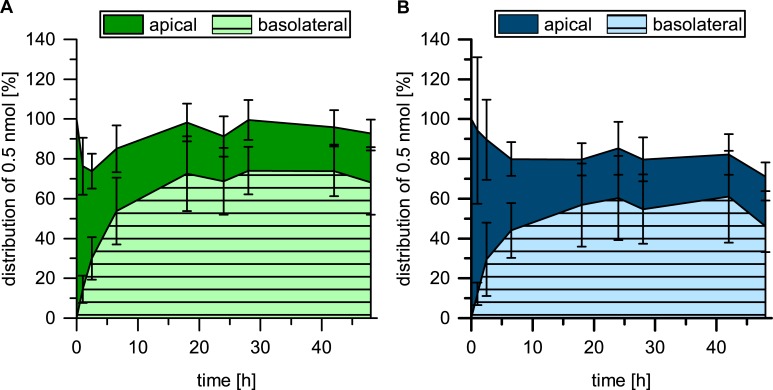
**Passive transfer kinetics of 0.5 nmol (1 μM apical application) of ENN B (A) and ENN B1 (B) through the PBCEC monolayer over the course of 48 h (*n* = 9)**. Concentrations recovered in the apical and basolateral compartment at every sampling time-point are displayed as relative amount of the initially applied amount of test substance (here: 0.5 nmol ENN B (A) and ENN B1 (B)). Data are presented as the mean ± standard deviation (SD).

Samples were drawn from the apical (“blood”-side) and basolateral (“brain”-side) compartment of the PBCEC two-compartment model after 1, 2.5, 6.5, 18, 24, 28, 42 and 48 h and quantitated via LC-MS/MS. The results are summarized in Fig 5. Already after 6.5 h 53% of the apically applied ENN B were found in the basolateral compartment soon reaching a plateau at 70 ± 4% (Fig 5A). Along with the quantitation in the apical and basolateral cell culture medium, the polycarbonate filter membranes with the attached PBCEC monolayers were extracted and analyzed after 48 h of incubation (data not shown). Even though ENN B was detectable in the filter membranes, the amount in relation to the initially applied amounts was below 1% and therefore negligible in terms of contributing to the overall distribution of ENN B in the system. After completion of the experiment (48 h), the amount of ENN B recovered from the system, i.e. amount of compound in the apical and basolateral compartment summed up, is 92,8 ± 23,1% of the initially introduced amount of ENN B (0.5 nmol). The permeability coefficient *p*_*c*_ was calculated according to Eqs 1 and 2. For ENN B the obtained *P*_*e*_ (ENN B) is (14.9 × 10^−6^ cm/s). For ENN B1, a very similar transport kinetic was observed. ENN B1 also showed a very fast and high transfer from the apical to the basolateral compartment with a very similar derived permeability coefficient of *P*_*e*_ (ENN B1) = 14.6 × 10^-6^ cm/s. The permeability of PBCEC for ENN B and ENN B1 ranges in the same order of magnitude as the high apparent permeability compounds caffeine (*P*_*app*_ = 15.5 × 10^−6^ cm/s) and diazepam (*P*_*app*_ = 12.7 × 10^−6^ cm/s) [29], which are known to reach the brain. After 6.5 h 44% of the apically applied ENN B1 was detected in the basolateral compartment (Fig 5B). From 6.5 h to the completion of the experiment after 48 h, the applied 0.5 nmol ENN B1 in the apical compartment were transferred to the basolateral compartment up to 55%. Analysis of the polycarbonate filters including the attached PBCEC revealed that for ENN B1 2.7% of the initially applied amount of compound could be recovered. This could give a hint why the sum of ENN B1 in both compartments did not completely add up to 100% of the originally introduced amount of ENN B1 (0.5 nmol) but only 73,7 ± 20,2% after 48 h. To exclude the involvement of metabolization, a screening was carried out via high resolution mass spectrometry, but no metabolites were detected (data not shown).

To study whether there was any active transport, ENN B and ENN B1 were applied on both sides of the PBCEC barrier in equimolar concentrations of 200 nM for each toxin. This concentration did not have any barrier impairing effect on the PBCEC monolayer. For both compounds, ENN B and ENN B1, no conclusive results concerning active transport could be obtained (data not shown). Still, a minor enrichment in the apical compartment for both mycotoxins could be suggested.

After completion of each transport experiment, both active and passive, lucifer yellow (LY) was applied in 50 μM concentration in the apical compartments. Samples were drawn after 1 h and the amount of LY in the basolateral compartment was quantitated via external calibration. The permeability coefficients for LY through cells treated with the solvent control (ACN 0.1% and 0.02%) and through cells treated with the test compounds for the past 48 h were calculated (Table 2). The permeability coefficients range in the same order of magnitude for test substance treated cells and cells incubated with solvent, which is considerably lower than the permeability coefficients derived for ENN B and ENN B1 and indicates an intact and tight monolayer with low paracellular transfer of compounds.

**Table 2:** Permeability coefficients of the PBCEC monolayer to ENN B, ENN B1 and LY.

Analyte	Permeability coefficient [10^−6^ cm/s]	Analyte	Permeability coefficient [10^−6^ cm/s]
ENN B	14.9	ENN B1	14.6
LY_(ENN B, passive)_	1.9	LY_(ENN B, active)_	0.7
LY_(ENN B1, passive)_	2.2	LY_(ENN B1, active)_	0.9
LY_(ACN 0.1%)_	3.2	LY_(ACN 0.02%)_	0.2

Derived permeability coefficients (*n* > 3) for the permeation of ENN B and ENN B1 from the apical to the basolateral compartment through the PBCEC monolayer for 1 μM apical application (passive transport). Permeability coefficients for LY from the apical to the basolateral compartment through the PBCEC monolayer after treatment with 1 μM ENN B and ENN B1 apically (passive transport), 200 nM ENN B and ENN B1 equimolar in both compartments (active transport) and the corresponding negative controls (ACN 0.1% and ACN 0.02%), respectively.

ENNs are able to penetrate different barriers in higher organisms. Keeping this in mind, in pigs, ENN B1 is quickly absorbed after oral uptake (*k*_a_ = 4.66 h^-1^_,_ *T*_max_ = 0.24 h) with a very high oral bioavailability of up to 91%. This is even higher than expected from a previous *in vitro-*study, performed via an intestinal uptake experiment with Caco-2 cells. The bioavailabilities for ENN B1 are suggested to be around 55 to 66% [13, 30]. In the applied BBB model, a very fast transfer of ENN B and B1 from the “blood”-side (apical) to the “brain”-side (basolateral) is shown. In mice, the influx of ENN B and ENN B1 shows rapid initial influx rates with a brain tissue distribution of 95% in the parenchyma and only 5% of the ENNs remaining in the capillaries [14]. No metabolism was observed in the Caco-2 model (*in vitro*), an *in vivo-*study in mice and the applied porcine BBB model described in this study [13, 14, 30]. In general, metabolization of these mycotoxins is described in other models. In liver microsomes of various origins (human, dog and rat), several phase I metabolites of ENN B are formed, hence phase I enzymes like CYP3A and CYP1A play a key role in the metabolism of this mycotoxin [31]. In pigs an assortment of ENN B and ENN B1 phase I-metabolites was detected and also a correlation between administration route and formation of certain metabolites was found, indicating a pre-systemic metabolism of ENN B1 after oral administration [32].

## Conclusion

In this study, the transfer of ENN B and ENN B1 across the BBB and effects on cells of the BBB were evaluated. PBCEC present a reliable and well-established two-compartment *in vitro-*model mimicking the BBB. Although this model system cannot completely reflect the conditions in the complex and dynamic milieu of a human brain *in vivo*, it serves as good screening tool. The obtained findings, in combination with an array of toxicologic (*in vitro*) test systems, are crucial for providing valuable information for risk assessment concerning potential neurotoxic effects of food contaminants like mycotoxins.

The passive transport studies showed a high permeation of ENNs from the “blood”-side to the “brain”-side, whereas the active transport suggested only a weak efflux of ENNs from the basolateral to the apical compartment. As soon as ENNs come in contact with the BBB or even cross it, effects on different cells of the BBB have to be considered. ENN B and ENN B1 induced high cytotoxicity in the astrocytoma cell line CCF-STTG1. Within the evaluated concentration range and time of exposure, ENNs, especially ENN B, caused the induction of apoptosis rather than necrosis in CCF-STTG1 cells. While the effects on viability in microvascular endothelial cells of the brain were less pronounced than in the astrocytoma cells (CCF-STTG1).

Human exposure and the lack of toxicity data for full risk assessment of these mycotoxins, support the need for further evaluation of their underlying mechanism of action. Furthermore, the discrepancy between the high activity of cyclohexadepsipeptides *in vitro* and the “missing” effects *in vivo* has to be clarified.
